# Study and Optimization of a High-Performance SPR-PCF Temperature Sensor for Low-Temperature Monitoring Applications

**DOI:** 10.3390/mi17060679

**Published:** 2026-05-30

**Authors:** Xinyuan Wang, Ke Jia, Zixi Fu, Yifan Feng, Jingheng Xiao, Yulin Wang, Wenjiang Ye

**Affiliations:** 1 Department of Applied Physics, Hebei University of Technology, Tianjin 300401, China; 235557@stu.hebut.edu.cn (X.W.); 235537@stu.hebut.edu.cn (K.J.); 18062889555@163.com (Z.F.); 18202573015@163.com (J.X.); 18797184231@163.com (Y.W.); 2 School of Mechanical Engineering, Hebei University of Technology, Tianjin 300401, China; 13722436668@163.com

**Keywords:** photonic crystal fiber, surface plasmon resonance, low-temperature sensing, mode-field control, finite element method

## Abstract

To meet the demand for highly sensitive temperature sensing in low-temperature environments, a surface plasmon resonance photonic crystal fiber (SPR-PCF) sensor with a central air hole and a dual-layer air-hole arrangement is designed and optimized. In this work, these air-hole features are used for mode-field regulation in a low-temperature sensing structure based on surface plasmon resonance (SPR), together with a polished gold film and an ethanol/chloroform (1:1) temperature-sensitive medium. The finite element method (FEM) was employed to analyze the resonance behavior and thermal response, and key structural parameters, including gold-film thickness, air-hole sizes, and radial positions, were optimized through cumulative parametric scanning. The optimized sensor shows good temperature response from −25 °C to 40 °C, with a maximum sensitivity of 36 nm/°C, a full width at half-maximum (FWHM) of 18.57 nm, and a figure of merit (FOM) of 1.2923. It is promising for cold-chain monitoring, low-temperature storage and transportation, and low-temperature sensing.

## 1. Introduction

Rooted in photonic bandgap theories [1,2], photonic crystal fiber (PCF) has revolutionized waveguide technology [3,4,5], enabling advanced light control via solid-core index guiding [6,7,8] and hollow-core bandgap guiding [9]. Compared with conventional optical fibers, PCF allows precise control of mode-field area [10], dispersion [11], and birefringence [12]. These properties enhance evanescent-field interactions and make PCF attractive for optical sensing [13]. PCFs have also promoted nonlinear optical studies, such as supercontinuum generation and nonlinear pulse transmission [4,5,8], and supported the development of advanced fiber-optic sensors [13].

Over the past few years, sensors based on SPR have attracted considerable attention for their highly sensitive temperature and refractive index sensing [14,15,16,17,18,19,20,21,22,23]. Previous studies have improved sensing performance by optimizing fiber geometry, tuning air-hole dimensions and spacing, and introducing functional materials. For instance, dual-parameter SPR-PCF sensors for temperature/refractive index [18] and temperature/magnetic field sensing [19] have been reported. Furthermore, significant progress has been made in enhancing temperature sensitivity. Han et al. [20] reported a liquid-filled hollow-core negative-curvature fiber sensor with a temperature sensitivity of 2.860 nm/°C over 20~40 °C. Wang et al. [21] developed a D-shaped, polished SPR-PCF sensor with a maximum sensitivity of 6.36 nm/°C over the temperature range of −5 °C to 60 °C. Li et al. [23] further increased the sensitivity to 15.4 nm/°C over the temperature range from −10 °C to 60 °C by enabling direct contact of the gold layer with the temperature-sensitive medium.

However, targeted designs of SPR-PCF temperature sensors for low-temperature monitoring remain limited. Most reported sensors focus on room-temperature or wide-range temperature detection, while the role of PCF geometry in low-temperature sensing has not been fully emphasized. Since the resonance response is governed by both the thermo-optic properties of the liquid medium and the coupling between the core mode and the surface plasmon polariton (SPP) mode, an optimized fiber structure is required to enhance modal overlap and improve spectral distinguishability in the low-temperature range.

In this work, a high-sensitivity SPR-PCF temperature sensor is proposed for low-temperature monitoring. The main contribution of this study is the design of a circular PCF structure with a central air hole and a dual-layer air-hole arrangement. This structure is used to reshape the effective core region, regulate the modal-field distribution, and improve phase matching between the core mode and the SPP mode. The resonance characteristics and temperature response are analyzed using FEM, and key structural parameters, including the gold-film thickness, air-hole diameters, and radial positions, are optimized through cumulative parametric scanning.

## 2. Proposed Sensor Design, Fabrication, and Measurement Setup

**Figure 1:**
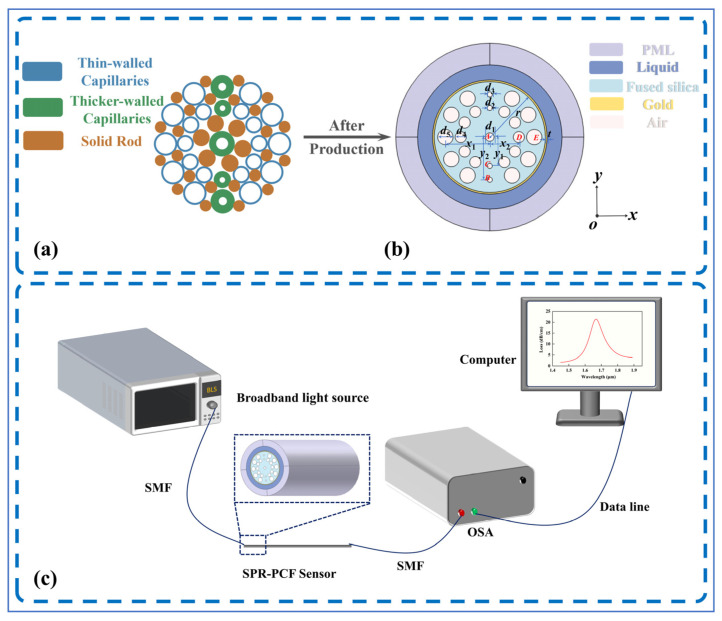
Overview of the proposed mode-field-controlled SPR-PCF temperature sensor, including (**a**) the preform design for fiber fabrication, (**b**) the cross-sectional geometry and simulation domain with key parameters, where A–E denote the different air-hole types, and (**c**) schematic view of a possible experimental measurement setup for resonance-wavelength tracking.

Figure 1 shows the design concept, cross-sectional structure, feasible fabrication route, and schematic measurement configuration of the proposed SPR-PCF temperature sensor. The cross-sectional model was established using two-dimensional FEM in COMSOL Multiphysics 6.3. The designed PCF can be fabricated by the stack-and-draw method. First, the preform is prepared according to Figure 1a. Thick-walled capillaries, thin-walled capillaries, and solid silica rods are assembled layer by layer to form the air-hole structure and core region. The thin-walled capillaries form the smaller air holes, while the thick-walled capillaries form the larger holes and provide mechanical support. The solid silica rods further improve the stability of the stacked structure. Then, the assembled preform is drawn in a standard fiber-drawing tower to obtain the desired PCF [24,25]. After fiber drawing, side polishing can be performed to expose a flat sensing plane on one side of the PCF. Subsequently, the gold layer is deposited on this polished surface by magnetron sputtering [18] or thermal evaporation. In this way, the metal film is coated on the external sensing plane rather than inside small air holes, making the coating process more accessible and controllable. Nevertheless, deviations in air-hole diameter, polishing depth, and gold-film thickness may still cause a shift in the resonance peak. In addition, careful axial alignment between the single-mode fiber (SMF) and PCF sensing section is required during measurement to reduce coupling loss.

Figure 1b illustrates the initial cross-sectional configuration of the proposed PCF sensor. The designed PCF consists of four parts: an outer perfectly matched layer (PML), a temperature-sensitive liquid layer, a fused-silica core, and an inner air-hole structure. The air-hole structure includes a central air hole A and two concentric rings of air holes symmetrically distributed about the *x*- and *y*-axes. Taking the center of air hole A as the origin O, a Cartesian coordinate system is defined, with the positive *x*-axis directed to the right and the positive *y*-axis directed upward. The central air hole A is positioned at the center of the fiber, with a diameter *d*_1_ = 1 μm, and is used to regulate the mode-field distribution and improve the coupling between the core mode and the SPP mode. The outer ring consists of 10 large holes and 2 small holes, which enhance mode confinement and suppress energy leakage into the cladding, thereby strengthening the SPR effect. The 10 outer large holes have a diameter of *d*_5_ = 1.8 μm and are uniformly distributed on a circle of radius *x*_1_ = 5.2 μm, while the 2 outer small holes have a diameter of *d*_3_ = 0.6 μm and are located on the *y*-axis at *y*_2_ = 5.0 μm. The inner ring also contains 10 large holes and 2 small holes to adjust the effective core size further and optimize the mode-field distribution. Their diameters are *d*_4_ = 1.3 μm and *d*_2_ = 0.6 μm, and their radial positions are defined by *x*_2_ = 3.4 μm and *y*_1_ = 3.4 μm, respectively. Therefore, the proposed design is characterized by a specific circular air-hole configuration and a parameterized combination of hole diameters and radial positions. The region outside the air holes is composed of fused silica with a radius of *r* = 6.5 μm. To enable temperature sensing, a gold nanofilm with a thickness of t = 29 nm is deposited on the polished cladding surface as the plasmonic excitation layer. The temperature-sensitive medium consists of a 1:1 ethanol/chloroform mixture with a large thermo-optic coefficient. Previous studies have shown that this mixture exhibits good temperature-response characteristics in fiber-optic temperature sensing [23]. In addition, the freezing points of ethanol and chloroform are approximately −114.1 °C and −63.5 °C, respectively, both of which are lower than −25 °C. Therefore, the mixture maintains good stability at low temperatures. Based on this, the temperature-sensitive medium used in this sensor is a 1:1 ethanol/chloroform mixture. In addition, a PML with a thickness of 2.55 μm is incorporated at the outermost boundary to absorb outwardly radiated electromagnetic energy and suppress numerical reflection, thereby improving computational stability and accuracy.

Figure 1c shows the experimental arrangement of the proposed temperature sensor. Light from a broadband source is launched into the SPR-PCF sensing section through an SMF. The output light is then guided out through another SMF and sent to an optical spectrum analyzer (OSA), and the data are transferred to a computer for real-time acquisition and processing through a data interface. During the measurement, an appropriate spectral range is selected to excite SPR, and the resonance wavelength associated with the loss peak is extracted at each temperature. By monitoring the shift in the resonance wavelength within the loss spectrum, the refractive-index variation in the temperature-sensitive medium can be inferred, thereby enabling temperature measurement.

## 3. Sensing Mechanism

### 3.1. Material Optical Models

Fused silica is adopted as the background material in the proposed SPR-PCF temperature sensor, and its refractive index can be determined using the Sellmeier Equation [26]:

(1) $$n^{2}(\lambda ,T)\;=\;\left(a_{0}+a_{1}T\right)+\frac{\left(b_{0}+b_{1}T\right)\lambda^{2}}{\lambda^{2}-\left(c_{0}+c_{1}T\right)}+\frac{\left(f_{0}+f_{1}T\right)\lambda^{2}}{\lambda^{2}-e}$$

where *a*_0_ = 1.31552, *a*_1_ = 6.90754 × 10^−6^, *b*_0_ = 0.788404, *b*_1_ = 2.35835 × 10^−5^, *c*_0_ = 0.0110199, *c*_1_ = 5.84758 × 10^−7^, *f*_0_ = 0.91316, and *f*_1_ = 5.48368 × 10^−7^, *e* = 100. The incident wavelength *λ* is expressed in micrometers (μm), and the temperature T is in degrees Celsius (°C). The sensor’s operating temperature range is −25 °C to 40 °C. Since the melting point of fused silica is 1670 °C, the influence of this temperature range on the fiber background material’s properties can be approximately neglected [23].

A gold layer is deposited on the polished surface of the fiber, and its dielectric constant is described using the Drude–Lorentz model [27]:

(2) $$\varepsilon_{\mathrm{Au}}\;=\;\varepsilon_{\infty }-\frac{\omega_{D}^{2}}{\omega (\omega +i\gamma_{D})}-\frac{\Delta \varepsilon \Omega_{L}}{(\omega^{2}{-\Omega }_{L}^{2})+i\Gamma_{L}\omega }$$

where ε_Au_(*ω*) represents the dielectric constant of gold at angular frequency *ω*, and *ε*_∞_ denotes the high-frequency dielectric constant limit, with ε_∞_ = 5.9673. The relationship between angular frequency and incident wavelength is expressed as *ω* = 2πc/*λ*, where c = 3 × 10^8^ m/s is the speed of light in vacuum and λ is the incident wavelength. In the Drude term, the plasma frequency and damping frequency are taken as ω_D_/2π = 2113.6 THz and γ_D_/2π = 15.92 THz, respectively. In the Lorentz term, the weighting factor, broadening parameter, and oscillator strength are set to Δε = 1.09, Γ_L_/2π = 104.86 THz, and Ω_L_/2π = 650.07 THz, respectively.

The temperature-sensitive medium consists of a 1:1 ethanol/chloroform mixture, and its refractive index is expressed as follows [21]:

(3) $$n\;=\;x\%\times \left\{[{n_{\mathrm{chloroform}}]}_{T=20\;{}^{\circ}C}+\frac{dn_{\mathrm{chloroform}}}{dx}\times \left(T-20\right)\right\}+(1-x\%)\times \;\left\{[{n_{\mathrm{ethanol}}]}_{T=20\;{}^{\circ}C}+\frac{dn_{\mathrm{ethanol}}}{dx}\times \left(T-20\right)\right\}$$

where *x*% and (100 − *x*)% denote the volume fractions of ethanol and chloroform, respectively, and d*n*/d*T* is the thermo-optic coefficient of each component. The thermo-optic coefficients of ethanol and chloroform are −3.94 × 10^−4^/°C and −6.328 × 10^−4^/°C, respectively. In this study, the dispersion of the temperature-sensitive liquid is neglected. At 20 °C, the refractive indices of ethanol and chloroform are 1.36048 and 1.43136, respectively, and the two liquids are mixed in a 1:1 volume ratio. Since the boiling points of ethanol and chloroform are 78.4 °C and 61.3 °C, respectively, the suitable operating temperature for the mixture should be below 60 °C [23]. To ensure medium stability, the operating temperature range of the proposed sensor is set to −25 °C to 40 °C.

### 3.2. Resonance Principles

The operating mechanism of the proposed temperature sensor is described as follows. Incident light is confined within the PCF core through total internal reflection, giving rise to an evanescent field near the gold layer. This evanescent field can induce collective oscillations of free electrons on the metal surface, thereby exciting SPR when the phase-matching condition is satisfied. At the resonance wavelength, the energy of the core mode is strongly coupled to the SPP mode at the metal interface, resulting in enhanced core loss and a pronounced loss peak in the confinement loss (CL) spectrum. Therefore, the CL of the core-guided mode is adopted to characterize the excitation strength of SPR, and it can be derived from the imaginary part of the effective refractive index as follows [28]:

(4) αloss(dB/cm) = 8.686×2πλ×Im(neff)×104

where Im(*n*_eff_) denotes the imaginary part of the effective refractive index of the incident mode, and *λ* represents the incident wavelength. The CL in Equation (4) is determined by the imaginary part of the effective refractive index, which represents the attenuation of the guided mode during propagation. When the core mode is phase-matched with the SPP mode, more optical energy is transferred from the core region to the gold/liquid interface. As a result, Im(*n*_eff_) increases, and a distinct loss peak appears in the CL spectrum.

As the temperature varies, the loss peak correspondingly shifts. Therefore, the sensing performance can be further evaluated by monitoring the resonance-wavelength shift in the CL spectrum under different temperatures. In this work, the wavelength sensitivity and FOM are selected as the main performance metrics of the temperature sensor. These two parameters are defined by Equations (5) and (6), respectively [29]:

(5) $$\mathrm{Sensitivity}\;(\mathrm{nm}/{}^{\circ}C)=\frac{\Delta \lambda_{\mathrm{peak}}}{\Delta T}$$

(6) $$\mathrm{FOM}\;({{}^{\circ}C\;}^{-1})=\frac{\mathrm{Sensitivity}}{\mathrm{FWHM}}$$

where sensitivity (nm/°C) refers to the wavelength sensitivity and is defined as the ratio of the resonance-wavelength shift Δ*λ* to the temperature variation Δ*T*. A larger sensitivity indicates a more pronounced wavelength response to a unit temperature change and thus a higher temperature resolution. Based on the above definition, FOM reflects both resonance-peak sensitivity and FWHM. A high sensitivity alone may not ensure good sensing performance if the resonance peak is too broad. Therefore, a larger FOM indicates a sharper resonance peak, more accurate peak identification, and better distinguishability of small wavelength shifts.

Physically, the resonance response is governed by the overlap between the evanescent field of the core mode and the SPP mode at the gold/liquid interface. A stronger modal overlap facilitates energy transfer from the core mode to the SPP mode and produces a more pronounced loss peak. In addition, since the refractive index of the liquid changes with temperature, the SPP mode is modified accordingly, leading to a resonance-wavelength shift. Therefore, the field distribution near the sensing interface is directly related to the temperature sensitivity and FOM.

## 4. Resonance Characteristics and Parameter Optimization of the Proposed Sensor

**Figure 2:**
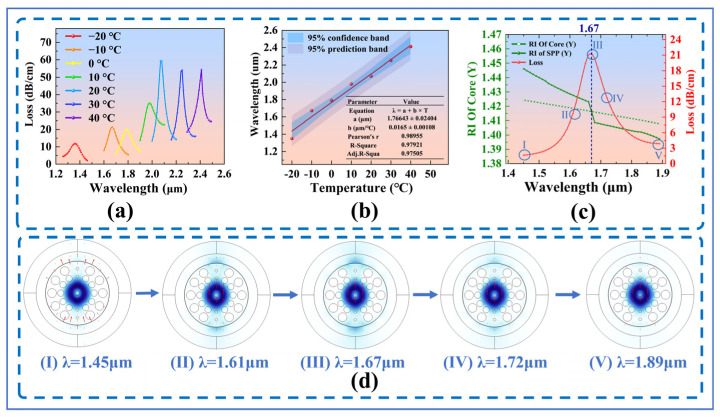
Resonance characteristics of the unoptimized SPR-PCF design: (**a**) Y-pol core-mode loss spectra versus temperature from −20 °C to 40 °C; (**b**) linear fit with the red fitting line of resonance wavelength versus temperature from −20 °C to 40 °C; (**c**) Y-pol loss spectrum and dispersion of the Y-pol core mode and SPP mode at T = −10 °C; (**d**) Y-pol electric-field distributions at representative wavelengths along the wavelength-evolution direction at T = −10 °C.

Figure 2 presents the structure of the unoptimized SPR-PCF and its resonance characteristics. Figure 2a shows the confinement-loss spectra of the Y-polarized (Y-pol) core mode over the temperature range from −20 °C to 40 °C, in steps of 10 °C. A distinct resonance-loss peak appears at each temperature. As the temperature increases, the CL spectra shift toward longer wavelengths, and their intensities also change. Figure 2b further illustrates the relationship between resonance wavelength and temperature, together with the linear fitting result and the 95% confidence and prediction bands. The fitting result indicates a clear positive linear correlation between resonance wavelength and temperature, with a coefficient of determination (R^2^) of 0.97921. Figure 2c depicts the dispersion relationship between the Y-pol core mode and the SPP mode. As shown in the enlarged view, phase matching occurs when the effective refractive indices of the two modes become equal, at which point the loss reaches its maximum and the corresponding resonance wavelength is approximately 1.67 μm. Figure 2d further displays the electric-field distributions at several characteristic wavelengths. Far from the resonance condition, the electric field is primarily confined within the core region. Near resonance, the effective indices of the core mode and SPP mode become closer. The field then extends from the core region toward the gold/liquid interface, enhancing modal overlap and energy transfer. This stronger coupling leads to increased confinement loss at the resonance wavelength.

The positions and dimensions of the air holes, together with the gold-layer thickness, affect the coupling strength between the core mode and the SPP mode, thereby influencing the resonance wavelength, loss-peak profile, sensitivity, FWHM, and FOM. Therefore, to improve the sensing performance of the SPR-PCF temperature sensor, a cumulative parametric scanning approach was adopted. Considering the possible coupling effects among different structural parameters, each parameter was optimized based on the previously optimized structure, rather than by independently selecting and simply combining individual optimum values. Based on the initial fiber structure, the sensing performance at −20 °C was taken as the evaluation criterion, and the parameters *t*, *y*_1_, *y*_2_, *x*_1_, *x*_2_, and *d*_1_~*d*_5_ were optimized sequentially. The temperature of −20 °C was selected as a representative low-temperature point for parameter comparison, and the optimized structure was further evaluated over the whole operating temperature range.

**Figure 3:**
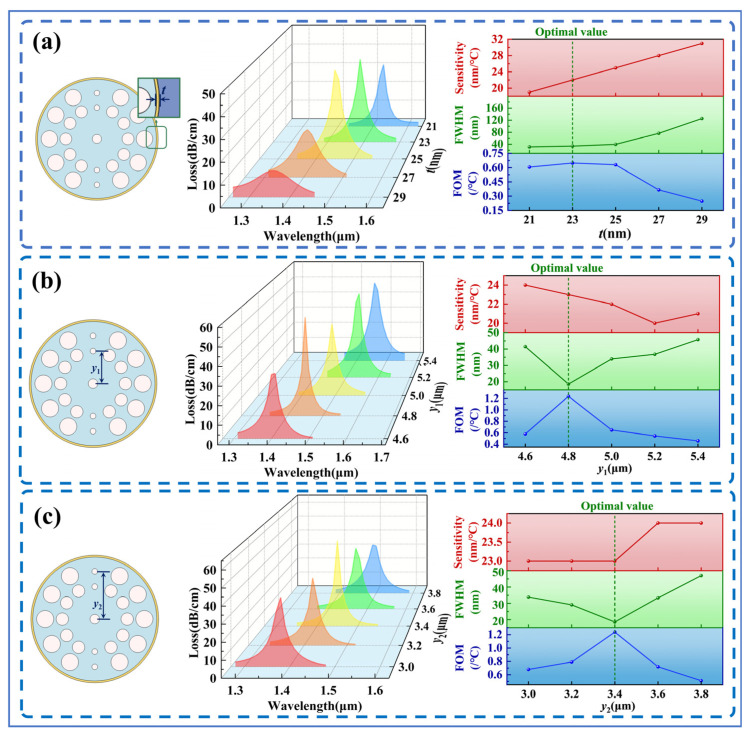
Parametric optimization of the SPR-PCF at −20 °C for (**a**) gold film thickness *t*, (**b**) inner-hole radial distance *y*_1_, and (**c**) outer-hole radial distance *y*_2_. For each sub-figure, the **left**, **middle**, and **right** panels represent the cross-sectional schematic, the evolution of CL spectra with wavelength, and the dependence of sensitivity, FWHM, and FOM on the corresponding parameter, respectively. In the middle panels, the CL spectra shown in different colors correspond to different values of the corresponding structural parameter. The optimal parameters are *t* = 23 nm, *y*_1_ = 4.8 μm, and *y*_2_ = 3.4 μm.

The optimization results for *t*, *y*_1_, and *y*_2_ are presented in Figure 3. Figure 3a shows that variation in t alters the coupling strength between the core mode and the SPP mode, thereby affecting the resonance-peak profile and spectral width; a comprehensive comparison indicates that the optimal performance is obtained at *t* = 23 nm. Figure 3b,c show that *y*_1_ and *y*_2_ affect the coupling behavior by regulating the mode-field distribution and the phase-matching condition. When *y*_1_ = 4.8 μm and *y*_2_ = 3.4 μm, the FWHM is the smallest, the loss peak is the highest, and the FOM is the largest, and these values are therefore selected as the optimal values.

**Figure 4:**
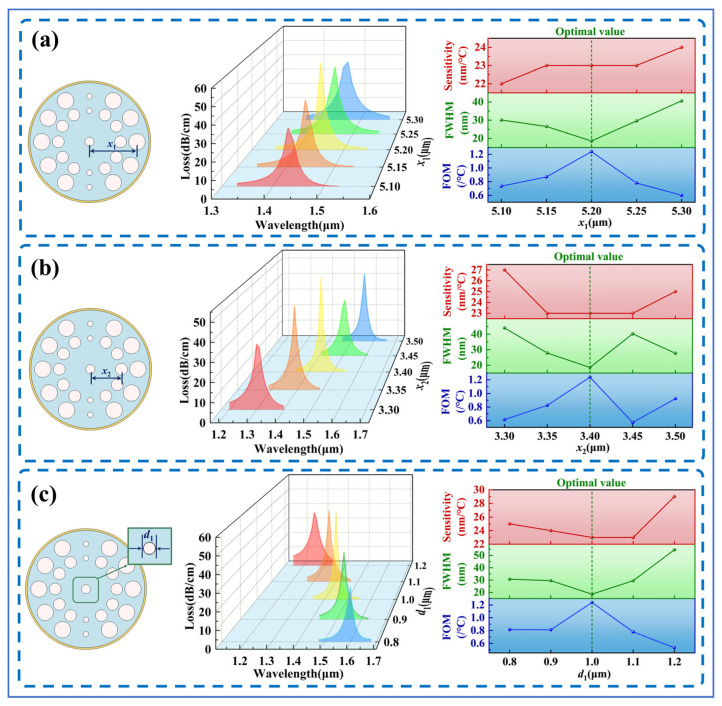
Parametric optimization of the SPR-PCF at −20 °C for (**a**) outer-layer radial distance *x*_1_, (**b**) inner-layer radial distance *x*_2_, and (**c**) central air-hole diameter *d*_1_. For each sub-figure, the **left**, **middle**, and **right** panels represent the cross-sectional schematic, the evolution of CL spectra with wavelength, and the dependence of sensitivity, FWHM, and FOM on the corresponding parameter, respectively. In the middle panels, the CL spectra shown in different colors correspond to different values of the corresponding structural parameter. The optimal parameters are *x*_1_ = 5.20 μm, *x*_2_ = 3.40 μm, and *d*_1_ = 1.0 μm.

The optimization results for *x*_1_, *x*_2_, and *d*_1_ are shown in Figure 4. *x*_1_ and *x*_2_ primarily influence the coupling between the core mode and the SPP mode by modifying the energy confinement in the cladding. As shown in Figure 4a,b, when *x*_1_ = 5.20 μm and *x*_2_ = 3.40 μm, the sensor maintains high sensitivity while exhibiting better spectral resolution, and these two values are therefore selected as the optimal parameters. As shown in Figure 4c, changing the diameter of the central air hole, d1, directly modifies the low-index region at the fiber center, thereby changing the effective refractive index and confinement of the guided core mode. A suitable central air hole weakens excessive core confinement and allows part of the modal field to extend toward the gold/liquid interface, which increases the overlap between the core mode and the SPP mode and improves the phase-matching condition. However, an excessively large *d*_1_ may over-perturb the core mode and reduce the balance between mode confinement and interface coupling. Therefore, the maximum FOM obtained at *d*_1_ = 1.0 μm indicates the best coupling state, and this value is chosen as the optimal one.

**Figure 5:**
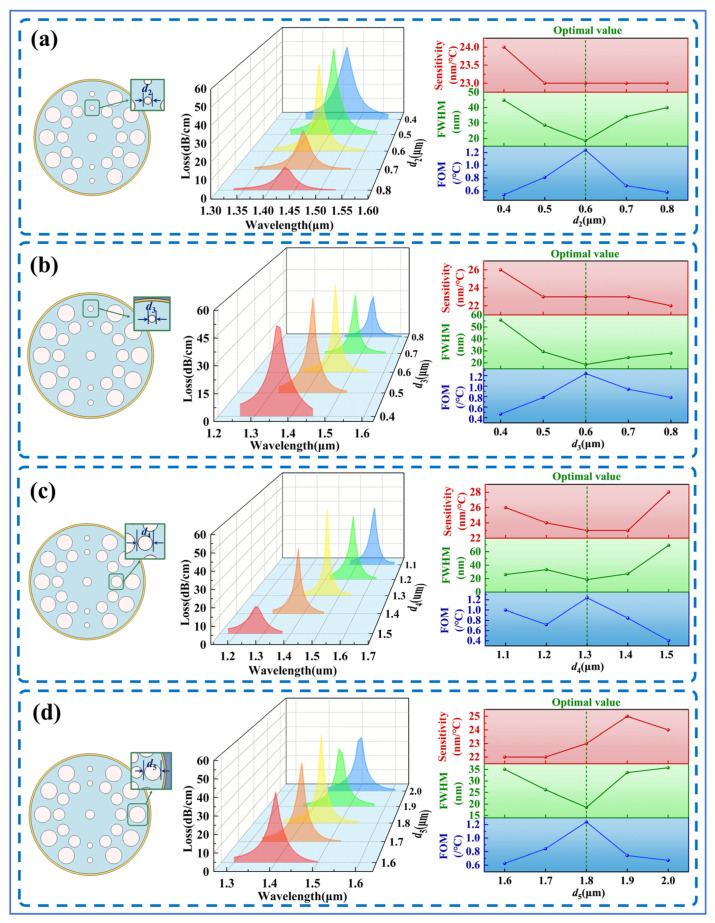
Parametric optimization of the SPR-PCF at −20 °C for (**a**) inner-layer small air-hole diameter *d*_2_, (**b**) outer-layer small air-hole diameter *d*_3_, (**c**) inner-layer large air-hole diameter *d*_4_, and (**d**) outer-layer large air-hole diameter *d*_5_. For each sub-figure, the **left**, **middle**, and **right** panels represent the cross-sectional schematic, the evolution of CL spectra with wavelength, and the dependence of sensitivity, FWHM, and FOM on the corresponding parameter, respectively. In the middle panels, the CL spectra shown in different colors correspond to different values of the corresponding structural parameter. The optimal parameters are *d*_2_ = 0.6 μm, *d*_3_ = 0.6 μm, *d*_4_ = 1.3 μm, and *d*_5_ = 1.8 μm.

The optimization results for *d*_2_, *d*_3_, *d*_4_, and *d*_5_ are shown in Figure 5. Variations in air-hole size change the effective refractive-index distribution in the core and cladding, thereby regulating the phase-matching condition between the core mode and the SPP mode. Physically, these air holes modify the mode-field area and the coupling channel between the fiber core and the gold/liquid interface. When the air-hole size is properly selected, more modal energy can participate in SPR coupling while the fundamental core mode remains well confined. Therefore, the changes in loss-peak intensity, FWHM, and FOM reflect the variation in coupling strength and field overlap caused by air-hole-size tuning. As shown in Figure 5a, with increasing *d*_2_, sensitivity decreases from a relatively high value and then becomes stable, while the FWHM first decreases significantly and reaches its minimum at the optimal value (*d*_2_ = 0.6 μm), where the FOM is also maximized. Figure 5b shows that, as *d*_3_ increases, sensitivity gradually decreases, while the FWHM first decreases and then slightly increases, reaching its minimum at *d*_3_ = 0.6 μm, where the FOM reaches its maximum, indicating a better phase-matching condition. In Figure 5c, variation in *d*_4_ causes the sensitivity to decrease first and then increase, whereas the FWHM reaches its minimum at *d*_4_ = 1.3 μm, corresponding to the maximum FOM, indicating that the mode-field distribution and coupling state are more favorable at this size. Figure 5d shows that, with increasing *d*_5_, sensitivity gradually increases, while the FWHM first decreases and then increases, reaching its minimum at *d*_5_ = 1.8 μm, where the FOM is the highest, indicating a better balance between mode confinement and coupling efficiency.

Through the stepwise cumulative optimization shown in Figure 3, Figure 4 and Figure 5, the optimal parameter combination of the sensor is determined. The gold-film thickness is set to *t* = 23 nm; the radial position parameters are *y*_1_ = 4.8 μm, *y*_2_ = 3.4 μm, *x*_1_ = 5.2 μm and *x*_2_ = 3.4 μm. In addition, the optimal air-hole diameters are *d*_1_ = 1.0 μm for the central hole, *d*_2_ = *d*_3_ = 0.6 μm for the inner and outer small holes, *d*_4_ = 1.3 μm for the inner large holes, and *d*_5_ = 1.8 μm for the outer large holes.

## 5. Performance Analysis of the Optimized SPR-PCF and Comparison

**Figure 6:**
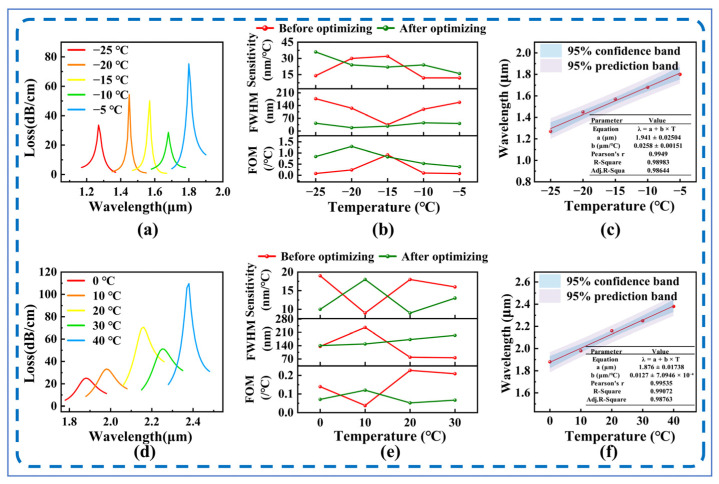
Temperature response of the optimized SPR-PCF sensor in the low-temperature and ambient-temperature regions. (**a**) CL spectra in the low-temperature range (−25 °C to −5 °C). (**b**) Comparison of sensitivity, FWHM, and FOM before and after optimization in the low-temperature range. (**c**) Linear fit of resonance wavelength versus temperature in the low-temperature range. (**d**) CL spectra in the ambient temperature range (0 °C to 40 °C). (**e**) Comparison of sensitivity, FWHM, and FOM before and after optimization in the ambient temperature range. (**f**) Linear fit of resonance wavelength versus temperature in the ambient temperature range.

After obtaining the optimized structural parameters, the sensor’s temperature response in the low- and ambient temperature regions was further analyzed, as shown in Figure 6. Figure 6a shows the variation in the CL spectra of the optimized sensor over the low-temperature range from −25 °C to −5 °C. As the temperature increases, the resonance peak shifts clearly, indicating a good temperature response at low temperatures. Figure 6b further compares the sensitivity, FWHM, and FOM before and after optimization in this region. The results show that structural optimization significantly reduces the FWHM and markedly improves the FOM. For example, at −20 °C, the FWHM decreases from 125.38 nm to 18.57 nm, while the FOM increases from 0.2393/°C to 1.2923/°C. At −25 °C, −10 °C, and −5 °C, the FOM also increases to 0.8412/°C, 0.5303/°C, and 0.3837/°C, respectively, indicating that the optimization substantially improves spectral resolution and sensing performance at low temperatures. Figure 6c presents the linear fitting result of resonance wavelength versus temperature in the low-temperature region, demonstrating good linearity and stable temperature sensing performance.

Figure 6d illustrates the variation in the CL spectra of the optimized sensor over the ambient temperature range from 0 °C to 40 °C. As the temperature varies, the resonance peak also shifts distinctly, indicating that the sensor maintains favorable temperature response characteristics in the ambient-temperature region. Figure 6e compares the sensitivity, FWHM, and FOM before and after optimization in this range. The results show that the optimized sensor still exhibits good temperature response and, to some extent, improved resonance-peak quality and overall sensing performance. Figure 6f shows a linear fit of the resonance wavelength versus temperature, indicating a stable wavelength response at ambient temperatures.

Table 1 compares the performance of the proposed sensor with those of previously reported simulation-based SPR-PCF temperature sensors. As shown, References [21,22,23] achieved relatively wide operating temperature ranges, but their maximum temperature sensitivities and FOM values are lower than those of the proposed sensor. For example, Reference [21] covers a temperature range of −5–60 °C, but its maximum sensitivity and FOM are 6.36 nm/°C and 0.344/°C, respectively. Reference [23] also provides a wide range of −10–60 °C, with a sensitivity of 15.4 nm/°C and an FOM of 0.2829/°C. In contrast, the proposed circular PCF sensor focuses on low-temperature monitoring and achieves a maximum sensitivity of 36 nm/°C and a maximum FOM of 1.2923/°C. This comparison indicates that, although some reported sensors provide wider detection ranges, the proposed structure shows better sensitivity and spectral distinguishability within the targeted low-temperature range.

## 6. Conclusions

In this study, an SPR-PCF temperature sensor based on a mode-field-controlled air-hole structure was designed and optimized for low-temperature detection by integrating a 1:1 ethanol–chloroform mixture with a polished gold film. The central air hole and dual-layer air-hole arrangement were used to regulate the modal field distribution and improve the coupling between the core mode and SPP mode.

Through cumulative parameter optimization, the optimized structure achieves a sharper resonance peak and improved sensing performance in the low-temperature range. The FEM results show a maximum temperature sensitivity of 36 nm/°C, a minimum FWHM of 18.57 nm, and a maximum FOM of 1.2923/°C. The reduced FWHM indicates improved spectral resolution, while the higher FOM suggests better distinguishability of small wavelength shifts.

These improvements result from both the high thermo-optic coefficient of the liquid medium and the geometry-induced mode-field control of the optimized SPR-PCF structure. Based on these results, the proposed design shows potential for high-resolution temperature monitoring in cold-chain transportation, low-temperature storage, and related low-temperature sensing scenarios.

## Figures and Tables

**Table 1:** Comparison of reported simulation-based SPR-PCF temperature sensors and this work.

Reference	Structure	Detection Range (°C)	Maximum Sensitivity (nm/°C)	Maximum FOM (/°C)
[18]	Indium tin oxide gold dual-core	10~50	12.3	0.3
[19]	Dual parameter	20~40	2.63	/
[20]	Liquid-filled hollow-core	20~40	2.86	/
[21]	D-shaped	−5~60	6.36	0.344
[22]	Ethanol toluene-filled	0~70	7.7	0.1169
[23]	Ethanol chloroform	−10~60	15.4	0.2829
This work	Circular PCF	−25~40	36	1.2923

## Data Availability

The original contributions presented in this study are included in the article. Further inquiries can be directed to the corresponding author.

## Abbreviations

The following abbreviations are used in this manuscript:

CL	Confinement Loss
FEM	Finite Element Method
FOM	Figure of Merit
FWHM	Full Width at Half-Maximum
OSA	Optical Spectrum Analyzer
PCF	Photonic Crystal Fiber
PML	Perfectly Matched Layer
SMF	Single-Mode Fiber
SPR	Surface Plasmon Resonance
SPR-PCF	Surface Plasmon Resonance Photonic Crystal Fiber
SPP	Surface Plasmon Polariton
Y-pol	Y-polarized
